# Tractography of the Spider Monkey (*Ateles geoffroyi*) Corpus Callosum Using Diffusion Tensor Magnetic Resonance Imaging

**DOI:** 10.1371/journal.pone.0117367

**Published:** 2015-02-18

**Authors:** Diana Platas-Neri, Silvia Hidalgo-Tobón, Benito da Celis Alonso, Fernando Chico-Ponce de León, Jairo Muñoz-Delgado, Kimberley A. Phillips

**Affiliations:** 1 Instituto Profesional de la Región Sur, Universidad Autónoma del Estado de Morelos, Jojutla, Morelos, Mexico; 2 Departamento de Neurocirugía, Hospital Infantil de México Federico Gómez, Mexico City, Distrito Federal, Mexico; 3 Departamento de Física, Universidad Autónoma Metropolitana, Iztapalapa, Mexico City, Distrito Federal, Mexico; 4 Facultad de Ciencias Físico-Matemáticas, Benemérita Universidad de Puebla, Puebla, Puebla, Mexico; 5 Dirección de Neurociencias, Instituto Nacional de Psiquiatría Ramón de la Fuente Muñiz, Mexico City, Distrito Federal, Mexico; 6 Facultad de Psicología, Universidad Nacional Autónoma de México, Ciudad Universitaria, Mexico City, Distrito Federal, Mexico; 7 Psychology Department, Trinity University, San Antonio, Texas, United States of America; University of Montreal, CANADA

## Abstract

The objective of this research was to describe the organization, connectivity and microstructure of the corpus callosum of the spider monkey (*Ateles geoffroyi*). Non-invasive magnetic resonance imaging and diffusion-tensor imaging were obtained from three subjects using a 3T Philips scanner. We hypothesized that the arrangement of fibers in spider monkeys would be similar to that observed in other non-human primates. A repeated measure (n = 3) of fractional anisotropy values was obtained of each subject and for each callosal subdivision. Measurements of the diffusion properties of corpus callosum fibers exhibited a similar pattern to those reported in the literature for humans and chimpanzees. No statistical difference was reached when comparing this parameter between the different CC regions (p = 0.066). The highest fractional anisotropy values corresponded to regions projecting from the corpus callosum to the posterior cortical association areas, premotor and supplementary motor cortices. The lowest fractional anisotropy corresponded to projections to motor and sensory cortical areas. Analyses indicated that approximately 57% of the fibers projects to the frontal cortex and 43% to the post-central cortex. While this study had a small sample size, the results provided important information concerning the organization of the corpus callosum in spider monkeys.

## Introduction

The corpus callosum (CC) is an organized system of fiber tracts that provides inter-hemispheric connectivity between the frontal, parietal, temporal and occipital cortices [1, 2]. This structure plays a central role in mediating complex behaviors and in the integration of hemispheric information between heterotopic and homotopic regions [3, 4].

In placental mammals, during brain development, the corpus callosum arises from dorsal commissures [5]. The growth of corpus callosum is associated with the reduction of the hippocampus and modifications of other structures, including the reduction of the anterior commissure, the elongation of the superior and inferior fibers of the fornix, and the expansion of the cerebral cortex [6, 5].

Among primates, the anterior region of the CC is linked to the marked expansion of the frontal lobe [7]. The tendency of brain volume to increase introduced potential connectivity problems for regions that are functionally linked but located in different anatomical hemispheres [8]. This is caused by a reduction of inter-hemispheric connections since the mid-sagittal area of the corpus callosum is smaller in primates with large brains [8]. Hemispheric dominance and lateralization phenomenon are correlated to increasingly independent hemispheres [8,9, 10]. In this context, the study of the tractography of the corpus callosum is fundamental in the knowledge of the evolution of lateralized structures and functions of the cerebral cortex [10, 11].

*Ateles* is one of the most extensive primate genera in America. Furthermore, it possesses one of the largest and most developed brain among the New World monkeys [12, 13]. The study of this genera has increased in recent years, but overall understanding of the *Ateles* evolution and ecology is based predominantly on behavioral and ecological studies. Even more, its neuroanatomy remains poorly understood [12, 14, 15, 16, 17, 18]. Spider monkey posses a brain volume of 101 cc, brain mass of 108 g and a body mass of 8000 g on average [12, 13]. Its gyri pattern is the most complex among cebids [12]. Moreover, this species has morphological and behavioral adaptations that have resulted from their arboreal life-form in highly dynamic systems like the rainforest [19, 20, 21] where they live in groups and feed through different foraging strategies [20, 22]. The body of the spider monkey bears four limbs, a long tail and long hands with a vestigial thumb that helps them move lightly through the forest canopy [22].

Recent techniques make possible a better reconstruction of corpus callosum. Diffusion is a three-dimensional process, and water molecular mobility in tissues is not necessarily the same in all directions. This diffusion anisotropy might result from the presence of obstacles that limit molecular movement in some directions. Slight anisotropic diffusion effects were observed in biological tissues during early studies, especially in tissues with strongly orientated components, such as excised rat skeletal muscles.

DTI is a non-invasive procedure that does not use exogen contrast. DTI builds indirectly high-resolution images of connections in the brain by detecting the diffusion of water molecules along axons [23, 24]. In white matter, the diffusion of water molecules does not follow a specific trajectory (anisotropy behavior) and therefore, is mainly caused by the orientation of the fiber tracts and by micro and macro structural characteristics of the white matter [24].

Diffusion tensor imaging is the more sophisticated form of Diffusion Weighted imaging, which allows for the determination of directionality as well as the magnitude of water diffusion. DTI enables to visualize white matter fibers in the brain and can map (trace image) subtle changes in the white matter associated with diseases such as multiple sclerosis and epilepsy, as well as assessing diseases where the brain’s wiring is abnormal, such as schizophrenia.

The properties of water diffusion are calculated along the X, Y, Z axis [24]. A color code is then assigned indicating the prevalent direction of water diffusion for each point. Fiber orientation on FA maps is highlighted by color coding, left-right uses red; anterior-posterior uses green; cranio-caudal uses blue [25].

The degree of diffusion of water molecules in white matter is described by fractional anisotropy (FA) parameter, caused mainly by the orientation of the fiber tracts, fiber density, axonal diameter and myelination [26]. FA gives information about the shape of the diffusion tensor at each voxel. The fractional anisotropy reflects differences between an isotropic diffusion and a linear diffusion.

Research on the evolution of the CC in non-human primates using diffusion tensor imaging (DTI) remains incipient [11, 27, 28]. Specifically, data of the corpus callosum analyzed by DTI in new world primates are inexistent. The few existing studies using DTI have focused on macaques and chimpanzees. One significant finding reported through electronic microscopy is that the proportional distribution of the different sizes of fiber in humans and macaques varies according to the region of the corpus callosum [1, 29, 30]. These studies have shown that the CC present similar topography in primates and that the CC contributes significantly the maintenance of complex cognitive abilities [11, 28, 29, 30, 31]. Nevertheless many questions still remain regarding the connections of fibers in the CC, during the evolutions of primate brain.

The study of the corpus callosum in spider monkey using DTI offers a greater comprehension of the evolution of inter-hemispheric brain connections in New World monkeys and primates in general. Diffusion tensor magnetic resonance imaging is a high sensitivity technique that enhances the possibility of obtaining quantitative information. The aim of this work was to elucidate the fiber organization of the corpus callosum in spider monkey. We present a description of the transcallosal fiber tracts connecting cortical regions as well as their magnetic resonance properties. Based upon recent work that describes the fiber tracts of the CC in other non-human primate species (rhesus macaques: [3, 28]; chimpanzees: [11]), we performed a series of experiments to test if the arrangement of fibers in spider monkey was similar to that observed in rhesus macaques and chimpanzees. This type of studies benefit and complement significantly non-human primate research as they provide useful structural, physiological and functional data [26].

## Methods

### Ethical statement

Research complied with protocols approved by Ethical committee of the National Institute of Psychiatry, the Mexican Official Norm NOM-062-ZOO-1999 and National Council for Science and Technology (CONACYT, project 109147), Mexico. In addition, the study followed the recommendations of The Weatherall Report for the use of non-human primates in research and the The ARRIVE Guidelines [32]. Specifically, subjects were socially housed in an outdoor enclosure (6 m in length, 6.3 m in height and 6.2 m wide), mean temperature 18.1°C, with artificial and natural stimuli, perches, and platforms. Individuals engaged in typical-species patterns of locomotion, including brachiation. Water and monkey chow were available *ad libitum*; fresh fruit and vegetables were provided daily.

### Subjects

Data was acquired from three healthy adult spider monkeys *(A. geoffroyi)* (female *n* = 2; male *n* = 1; mean age = 10.5 years ± SD 2.5 years; mean weight 10.9 kg ± SD 0.11). Subjects were kept in the animal housing facilities of the National Institute of Psychiatry, Ramón de la Fuente Muñiz, Mexico City. *At the beginning of the CONACYT project in 2010, this multimale-multifemale group consisted of four males and seven females. To avoid possible variations in the corpus callosum, the animals used in the experiment were the healthiest and the closest in age of the colony. At the end of this study, the animals were transferred to an outdoor enclosure, which is in the distributional range of the species*: *the primatological field station of Catemaco of the Universidad Veracruzana, Mexico*.

### Experimental protocol

During the morning, animals were anesthetized before being placed inside the scanner. Anesthesia was induced with an intramuscular injection of Ketamine (15 mg/kg, Pisa) and atropine (0.05 mg/kg, ABBOJECT) followed with Zoletil 50 Tiletamine-Zolasepan (0.2 mg/kg, Virbac); recording site was cleaned and sterilized with antibacterial sanitizer. A pediatric head immobilizer (Medihelp) was used to reduce the motion artifact. This immobilizer used plastic compartments filled with air to fixate the animal’s head, ensuring that head motion was in all cases less than 1.5 mm in all directions during the whole of the experimental time (50 minute procedure). Once the images were acquired, subjects were placed in a restriction cage for a period of 12 hours to allow for normal recovery. After this recovery period animals were reintroduced to the social group. During the image acquisition and recovery time, a veterinarian was present to monitor the physiological parameters and assess the well-being of the animals.

### Image acquisition

During the 50-minute procedure, anatomical images and DTI sequences were acquired form each subject. All MRI studies were performed on a 3T Achieva scanner (Philips Achieva, Best, Netherlands). This scanner was equipped with an eight-channel SENSE RF coil. A Quasar Dual gradient system was used with gradient amplitudes up to 80 mT/m and slew rates up to 200 mT/m/ms. An anatomical image set was acquired using a T_1_-3D-GE sequence with: TR = 10.6 ms, TE = 5.18 ms FOV: 150x150 mm, matrix: 256×256, 1 mm slice thickness and flip angle of 8°. DTI imaging was performed with an axial EPI sequence in 32 directions (b value 1000 s/mm^2^). Images presented a resolution of 2x2x2 mm; with a reconstructed voxel of: 0.78x0.78x2 mm. This sequence covered the same FOV as the anatomical sequence.

### Fiber-tracking and DTI image processing

Diffusion image analysis was performed using The Oxford Center for Functional Magnetic Resonance Imaging (FMRIB) software, FSL 3.2.0 (http://www.fmrib.ox.ac.uk/fsl) and MedINRIA 2.0.1 (http://medinria.fr) software.

Initial preprocessing included reorientation, removal of non-brain tissue and correction for head motion and eddy current distortion. FDT-DTIFIT was used to fit the diffusion tensor at each voxel and create FA maps. Each subject’s scan was registered to standard spider monkey space (created in-house) using a non-linear transformation (FNIRT) [33, 34, 35]. MedINRIA was then used to calculate diffusion tensors from all voxels. Fiber tracts were calculated by connecting adjacent voxels with similar principal eigenvectors, using a threshold FA value of 0.3 and a smoothness factor (a parameter ranging from 0 to 1 corresponding to the straightness of each fiber) of 0.2 for continuous fiber reconstruction. Fibers were limited to lengths larger than10 mm [33]. The fractional anisotropy threshold was set at 300 (corresponding to an actual FA value of 0.300), stopping FA threshold 200; minimum length of fibers 15 mm; smoothness at 20 and sampling at 1. The 3-dimensional structure was reconstructed for fiber tracts [36].

### Corpus callosum segmentation

**Fig 1:**
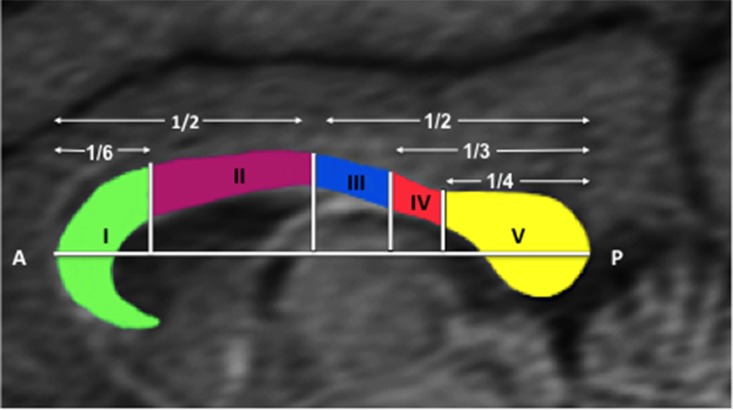
MR mid-sagittal image of the corpus callosum of an *Atles geoffroyi* monkey. Corpus callosum was divided in five regions according to the anatomic landmarks and Hofer ‘s method [3].

The process to determine the tractography of the entire corpus callosum was done according to the method suggested by Hofer [28] (Fig. 1). The area of the CC was manually traced in the midsagittal plane using the region of interest function (ROI) in MedINRIA. Additional ROIs were then traced, also from the midsagittal plane, to separate the transcallosal projections into five segments. This method divides the corpus callosum into subdivisions based upon the fiber projections to cortical regions: Rostrum and Genu I = prefrontal lobe, Anterior body II = premotor and supplementary motor cortices, Medium body III = primary motor cortex, Posterior body IV = primary sensorial cortex, Splenium V = parietal, temporal and occipital lobes.

We used the next anatomic landmarks [11, 12, 16, 17, 37, 38], to ensure a consistent identification of fiber bundles into cortical areas: 1) Prefrontal cortex: encompassed areas anterior to the arcuate sulcus. 2) The premotor and supplementary cortex were delimited in the anterior-posterior direction to arcuate sulcus and central sulcus. 3) The primary motor cortex was horizontally situated in the caudal part of the branch of arcuate sulcus and the anterior bank of the central sulcus. 4) The parietal cortex was located behind the central sulcus and parietooccipital sulcus. 5) The Temporal cortex was located bellow the lateral fissure and 6) Occipital cortex was situated behind inferior occipital sulcus and parieto-occipital sulcus.

### Statistical analysis

Data were analyzed using SPSS 21 (http://www-01.ibm.com/software/analytics/spss). Repeated measures (n = 3) of FA values were obtained of each subject and for each callosal subdivision (a mid-sagittal measure, 1mm lateral left and 1mm lateral right). As data failed normality, non-parametric tests such as Friedman for multiple comparisons and Mann–Whitney U tests for paired comparisons were used with a threshold of p = 0.05.

## Results

### Topography of the CC

**Fig 2:**
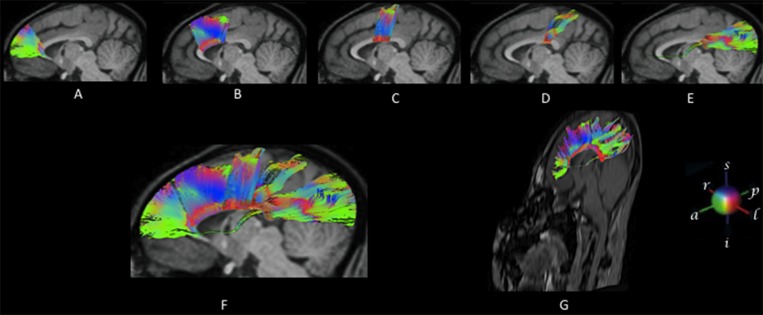
DTI results of a random subject of this study. Reconstructed fibers traversing the corpus callosum are over imposed on a mid-sagittal anatomical image. Fibers leaving the CC were divided in five groups (A-E). Rostrum and Genu I (2A), Anterior body II (2B), Medium body III (2C), Posterior body IV (2D), Splenium V (2E). All corpus callosum fibers are incorporated to views 2F (sagittal) and 2G (oblique). Color codes indicate direction. Red: left to right, green: anterior to posterior, blue: superior to inferior.

**Fig 3:**
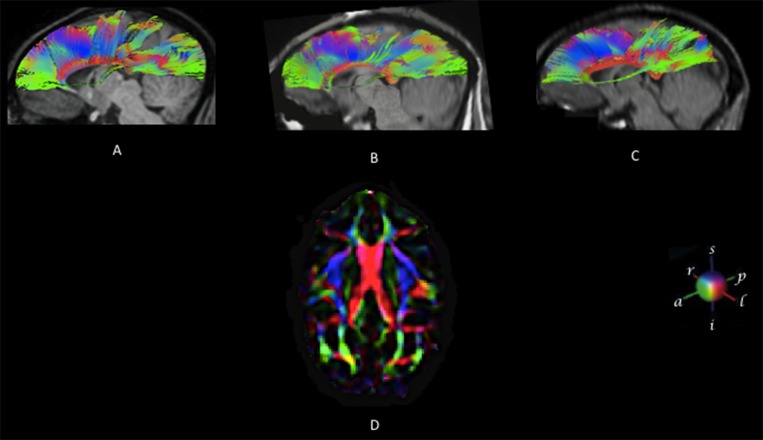
DTI results for n = 3 subjects. Reconstructed fibers traversing the corpus callosum are over-imposed on a mid-sagittal anatomical image (subject 1, 3A; subject 2, 3B; subject 3, 3C. Fig. 3D: The mean map of Axial Eigenvectors showing the principal diffusion tensor directions of CC in global orientation. Color codes: red: left to right, green: anterior to posterior, blue: superior to inferior.

The spatial distribution and order of fiber bundles depicted for a representative subject in Fig. 2 was observed to be similar to the arrangement found for the other two subjects, Fig. 3 (A-C). Fibers leaving the CC were divided in five groups as shown in Fig. 2 (A-E): Fibers associated with prefrontal cortex, (Fig. 2A); premotor and supplementary motor cortices, (Fig. 2B); primary motor cortex, (Fig. 2C); primary sensorial cortex, (Fig. 2D), parietal, temporal and visual cortical regions, (Fig. 2E) and all corpus callosum (Fig. 2F & 2G).

Using Witelson's geometric subdivision modified by Hofer’s [28, 39], the analysis of the fiber bundles areas indicated that approximately 18% of the fibers had projections to prefrontal cortex, 24% corresponded to the premotor and supplementary motor cortices, 15% to the primary motor cortex, 12% to the primary sensorial cortex and 31% projects to parietal, temporal and visual cortical regions. Regions I, II and III encompass all cortical projections into the frontal lobe and accounted for, across all subjects, approximately 57% of the length of the CC.

### Regional differences in FA

**Fig 4:**
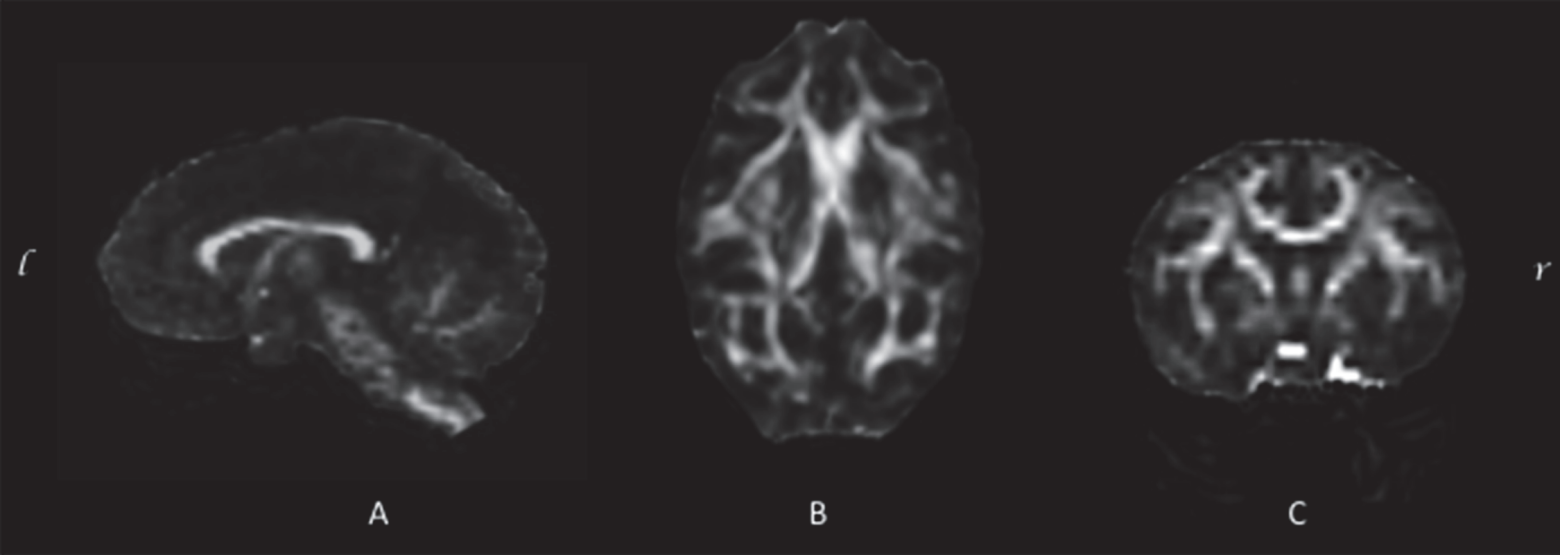
FA results. Orthogonal view displaying the mean Fractional Anisotropy (FA) maps from all subjects.

Fig. 3D shows a global view of an eigenvector color mean map for the three subjects. The map is an appropriate summary in which the degree of anisotropy and the global fiber directions can be determined. The corpus callosum stands out as a dominant red tract of fibers running orthogonal to the image plane; green tracts, such as the prefrontal and parietal, temporal and visual cortical regions, run primarily in the anterior-posterior direction. The color changes from green to blue (ascending-descending tracts) around the premotor and supplementary motor cortices (see also Fig. 2B & 2C). The mean FA value maps for all subjects is represented in Fig. 4.

**Fig 5:**
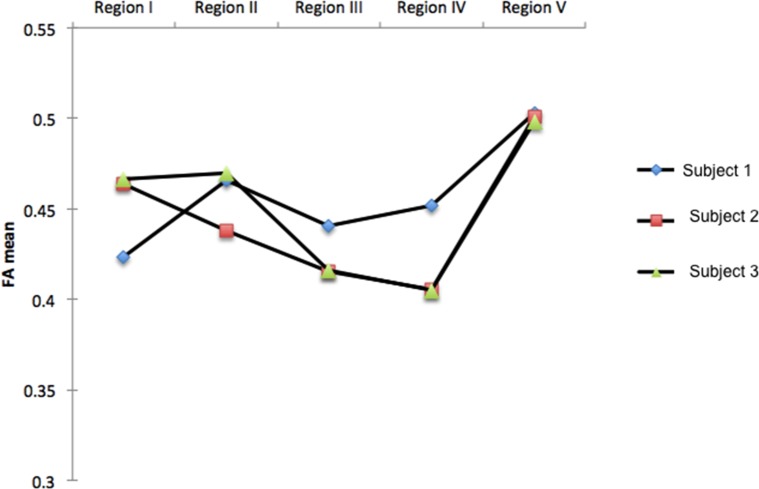
Regional FA comparison. Mean FA values for each subject and region.

When considering the FA values of each subject there was little variability observed between individuals (Fig. 5). Friedman test was conducted to evaluate differences within-subjects in FA among the 5 subdivisions of the corpus callosum. Although the analysis of diffusion properties showed no significant differences in FA (p = 0.066) (Table 1), measurements of the CC fibers exhibit a similar pattern to those reported in the literature for humans and chimpanzees (Table 2). The highest FA values were found in regions projecting to the visual and auditory cortex, also higher-association areas of posterior cortical (region V) and premotor and supplementary motor cortices (region II). The lowest FA values were seen in regions projecting into motor and sensory cortical areas (regions III and IV) (see Table 1).

**Table 1:** Mean FA value comparison.

FA		
Region	Mean	SD
I	0.451	0.024
II	0.457	0.017
III	0.424	0.014
IV	0.420	0.026
V	0.500	0.002

Mean FA values were calculated from the three measurements of the three subjects in each region in which CC was divided.

**Table 2:** Inter-species tractography comparison.

Specie	Percentage of callosal projection.		FA pattern per region	
	Frontal cortex	Postcentralcortex	Maximus value	Minimum value
*H. sapiens*	67%	33%	Region V, II, I	Region III, IV
*P. troglodytes*	64%	36%	Region V, II, I	Region III, IV
*M. mulatta*	60%	40%	Region II,III,I	Region IV, V
*A. geoffroyi*	57%	43%	Region V, II, I	Region III, IV

CC tractography results obtained in the literature for different species of primates (*H. sapiens* [3]; *P. troglodytes* [11]; *M. mulatta* [3, 28])

## Discussion

The objective of this research was to: First, analyze the tractography and FA values of the fibers of the corpus callosum of the spider monkey using DTI. Second, compare the layout and organization of the fibers in this type of monkey with those reported for other primates [3, 11, 28]. Based on these objectives, we hypothesized that the arrangement of fibers in spider monkeys would be similar to that observed in rhesus macaques, chimpanzees and humans. Due to the socio-ecological characteristics of spider monkeys, resulting from their arboreal life form in a highly dynamic environment such as the rainforest; we expected to find higher FA values in posterior regions involved in the visual processing of complex spatial tasks, the processing of auditory information such as detection of sound quality and the assembling of somatosensory system information.

Spider’s monkey fission-fusion social system (a system similar to that of chimpanzees and probably early humans [40, 41, 42] is characterized by the adjustment in the size of the group, where individuals cohere and are divided into subgroups according to the availability and distribution of resources [20, 42, 43, 44]. Due to the dynamic nature of these societies and the interaction with resources, these primates may have evolved under conditions that demand the development of visuospatial and communication skills and complex somatosensory integration [20, 42, 45, 46, 47].

The analysis of diffusion properties showed a consistent pattern in FA between regions, similar to those reported in other non-human primates [3, 11, 28]. The highest values in spider monkeys corresponded to regions projecting from the CC to the posterior cortical association areas, premotor and supplementary motor cortices and projections to the prefrontal lobe. The highest FA observed in spider might be a result of increased myelination and higher fiber packing density in parietal, temporal and occipital cortices, in response to the demands mentioned above.

Research in human post-mortem tissue shows that fibers with a large diameter (≥ 2 μm) are predominant in the middle regions connecting primarily motor, somatosensory and primary visual area, whereas fibers with a small diameter (≤ 2 μm) and a low concentration of myelinization are present in fibers from pre-frontal associated cortices and posterior regions connecting associative cortical areas [29,30]. These findings agree with the presence in our results of sets of fibers that are probably highly myelinized and with a large diameter in the primary visual (Region V), premotor and supplementary cortex areas (Region II) and with possibly medium-sized concentrations of myelinization with intermediate diameters in primary motor projections and somato-sensory areas (Regions III and IV). However, the evidence in prefrontal associated cortices of the reduction of levels of myelinization and fibers with a small diameter proposed by Aboitiz [29,30], does not coincide with the patterns observed in spider monkeys (Region I). This difference may be due to the fact that in studies with post-mortem material such as that proposed by Aboitiz [29,30], segmentation varies, particularly in the first third of the calloused body, in comparison with the segmentation followed in our study. However, it is important to simplify the complexity of the processing and the fiber composition in these areas. It is therefore necessary to supplement this information with histological and genetic coding studies for the regions indicated, and to undertake detailed studies incorporating anatomical cytoarchitectural parcellation and diffusion imaging tractography to achieve a fine correlation between inter-hemispheric brain connections and functional cytoarchitectures. [48].

### Organization and arrangement of the CC between different primates

Our data offers evidence in support of the notion that primates display similar topography of the CC [3, 11, 28]. Spider monkeys were shown to have 57% of callosal projections into the frontal cortex (encompassing regions I, II and III), and 43% in the postcentral cortex (IV and V). In humans, projections to the frontal cortex account for approximately 67% of callosal projections [3]. Chimpanzees have 64% of callosal projections in this region [11]. Rhesus monkeys have 60% of callosal projections to the frontal cortex [28] (Table 2).

Our DTI data supports the statement that evolutionary changes in brain volumes are reflected in the re-arrangement of related fibers crossing the corpus callosum [7, 28] and the fact that the anterior region of the CC is linked to the marked expansion of the frontal lobe [7], in conjunction with the evolutionary trend among primates to higher visual areas and the expansion of occipital and temporal [49, 50] cortices.

The degree of diffusion of water molecules in white matter is described by fractional anisotropy, caused mainly by the orientation of the fiber tracts, fiber density, axonal diameter and myelination [26]. Thus, high FA values should be observed in regions with densely distributed fibers with a relatively small diameter; while low values would be found in regions with a lower density of fibers and axons with a higher diameter [26]. Spider monkeys showed higher FA values in regions V, II and I and a decrease in the values in regions III and IV (Table 2). Although, FA values are higher in humans, chimpanzees and macaques, due to the relative increase in the volume of white matter. Results showed similar patterns for regions of CC in humans, chimpanzees and spider monkeys (Table 2).

While this study had a small sample size, the results provided important information concerning the organization of the CC and behavioral specialization in spider monkeys. We consider that the present study could be useful for future investigation on the evolution of inter-hemispheric brain connections in primates, since it is the first evidence of DTI, CC morphology in new world monkeys. We acknowledge that these results, particularly the observed regional differences in FA, must be replicated with a larger sample.

## Supporting Information

S1 Checklist ARRIVE Guidelines Checklist. (PDF)

S1 Table FA values from the three measurements of the three subjects in each region in which CC was divided. FA- Fractional anisotropy; Measure code: L = left; C = center; R = right.
(DOCX)
