# Correction: Tractography of the Spider Monkey (*Ateles geoffroyi*) Corpus Callosum Using Diffusion Tensor Magnetic Resonance Imaging

**DOI:** 10.1371/journal.pone.0123372

**Published:** 2015-03-30

**Authors:** The PLOS ONE Staff

The third author’s name is spelled incorrectly. The correct name is: Benito de Celis Alonso. The correct citation is: Platas-Neri D, Hidalgo-Tobón S, de Celis Alonso B, de León FC-P, Muñoz-Delgado J, Phillips K, et al. (2015) Tractography of the Spider Monkey (Ateles geoffroyi) Corpus Callosum Using Diffusion Tensor Magnetic Resonance Imaging. PLoS ONE 10(2): e0117367. doi:10.1371/journal.pone.0117367

## References

[1] Platas-Neri D, Hidalgo-Tobón S, da Celis Alonso B, de León FC-P, Muñoz-Delgado J, Phillips K, et al (2015) Tractography of the Spider Monkey (*Ateles geoffroyi*) Corpus Callosum Using Diffusion Tensor Magnetic Resonance Imaging. PLoS ONE 10(2): e0117367 doi:10.1371/journal.pone.0117367 25693078 10.1371/journal.pone.0117367 PMC4333290

