# Supplementary material for: Tractography of the Spider Monkey (Ateles geoffroyi) Corpus Callosum Using Diffusion Tensor Magnetic Resonance Imaging
Source: PLoS One. 2015 Feb 18;10(2):e0117367. doi: 10.1371/journal.pone.0117367 (PMC4333290; doi:10.1371/journal.pone.0117367)
Supplement: S1 Table — FA- Fractional anisotropy; Measure code: L = left; C = center; R = right. (DOCX) [file pone.0117367.s002.docx]

|  |  |  | **FA** |  |  |  |  |
| --- | --- | --- | --- | --- | --- | --- | --- |
| Subject ID Number | Sex | Number of measure | Region I | Region II | Region III | Region IV | Region V |
| 1T | M | 1L | 0.42366 | 0.46624 | 0.46061 | 0.45414 | 0.50378 |
|  |  | 2C | 0.41968 | 0.46332 | 0.43171 | 0.44702 | 0.50021 |
|  |  | 3R | 0.42580 | 0.46736 | 0.42938 | 0.45346 | 0.50368 |
| 2A | F | 1L | 0.44444 | 0.43938 | 0.41892 | 0.40678 | 0.50363 |
|  |  | 2C | 0.47417 | 0.43460 | 0.41459 | 0.41063 | 0.49915 |
|  |  | 3R | 0.47316 | 0.44056 | 0.41314 | 0.39865 | 0.49945 |
| 3M | F | 1L | 0.47021 | 0.46288 | 0.42926 | 0.39954 | 0.50113 |
|  |  | 2C | 0.43471 | 0.47832 | 0.42716 | 0.38653 | 0.49648 |
|  |  | 3R | 0.49648 | 0.46837 | 0.39315 | 0.43030 | 0.49703 |

Supporting Information

**S1 Table.** FA values.
